# Relationship between socioeconomic status and gastrointestinal infections in developed countries: a systematic review protocol

**DOI:** 10.1186/s13643-016-0187-7

**Published:** 2016-01-21

**Authors:** Tanith C. Rose, Natalie Adams, David C. Taylor-Robinson, Benjamin Barr, Jeremy Hawker, Sarah O’Brien, Mara Violato, Margaret Whitehead

**Affiliations:** NIHR Health Protection Research Unit in Gastrointestinal Infections, University of Liverpool, Liverpool, L69 7BE UK; NIHR Health Protections Research Unit in Gastrointestinal Infections, University of Oxford, Oxford, UK; Department of Public Health and Policy, University of Liverpool, Liverpool, UK; Institute of Infection and Global Health, University of Liverpool, Liverpool, UK; Public Health England, London, UK; Health Economics Research Centre, University of Oxford, Oxford, UK; Department of Public Health and Policy, Institute of Psychology, Health and Society, University of Liverpool, Whelan Building, Liverpool, L69 3GB UK

**Keywords:** Socioeconomic factors, Income, Social class, Employment, Education, Gastrointestinal infection, Diarrhoea, Gastroenteritis, Foodborne diseases

## Abstract

**Background:**

The association between low socioeconomic status (SES) and poor health is well documented in the existing literature. Nonetheless, evidence on the relationship between SES and gastrointestinal (GI) infections is limited, and the mechanisms underlying this relationship are not well understood with published studies pointing to conflicting results. This review aims to identify studies that investigate the relationship between SES and GI infections in developed countries, in order to assess the direction of the association and explore possible explanations for any differences in the risk, incidence or prevalence of GI infections across socioeconomic groups.

**Methods:**

Three systematic methods will be used to identify relevant literature: electronic database, reference list and grey literature searching. The databases MEDLINE, Scopus and Web of Science Core Collection will be searched using a broad range of search terms. Screening of the results will be performed by two reviewers using pre-defined inclusion and exclusion criteria. The reference lists of included studies will be searched, and Google will be used to identify grey literature. Observational studies reporting quantitative results on the prevalence or incidence of any symptomatic GI infections by SES, in a representative population sample from a member country of the Organisation for Economic Co-operation and Development (OECD), will be included. Data will be extracted using a standardised form. Study quality will be assessed using the Liverpool University Quality Assessment Tools (LQAT). A narrative synthesis will be performed including tabulation of studies for comparison.

**Discussion:**

This systematic review will consolidate the existing knowledge on the relationship between SES and GI infections. The results will help to identify gaps in the literature and will therefore provide an evidence base for future empirical studies to deepen the understanding of the relationship, including effective study design and appropriate data analysis methods. Ultimately, gaining insight into this relationship will help to inform policies to reduce any health inequalities identified.

**Systematic review registration:**

PROSPERO CRD42015027231

**Electronic supplementary material:**

The online version of this article (doi:10.1186/s13643-016-0187-7) contains supplementary material, which is available to authorized users.

## Background

There is strong evidence of a social gradient in most health outcomes whereby the poorest in society experience greater levels of illness and premature death than those further up the socioeconomic scale [1]. Socioeconomic inequalities are linked to both causes and consequences of ill health [2] and have been well documented in diseases of a non-infectious nature, such as coronary heart disease and cancer [3]. Whilst there is evidence that the incidence of many infectious diseases, such as tuberculosis and human immunodeficiency virus [4–6], varies by social group, the association between socioeconomic status (SES) and gastrointestinal (GI) infections in particular is not well understood.

Gastrointestinal infections, caused by organisms such as bacteria, viruses or protozoa, are a common source of disease in the UK, leading to diarrhoea and vomiting and potentially more serious health problems, all of which can interfere with normal daily life. Previous studies have estimated that around 25 % of people in the UK will suffer an episode of infectious intestinal disease (IID) per year and that foodborne illness (a proportion of IID) in England and Wales costs around £1.5 billion per annum [7, 8]. It is reported that 10 % of children present to healthcare services with gastroenteritis each year, accounting for 16 % of paediatric accident and emergency presentations in one study [9]. There are eight million absences from school and at least 11 million working days lost to the economy each year due to GI infections [7].

The impact of SES on vulnerability to GI infections is unclear, and the limited existing evidence points to conflicting results. Higher prevalence of GI infections is often thought to be associated with more advantaged individuals. However, a recent systematic review looking at the impact of SES on laboratory-confirmed foodborne illness in developed countries suggests that this relationship is not so clear [10]. Newman et al. [10] identified 16 studies across four pathogens with mixed results, differing by pathogen. For example, in the most disadvantaged populations compared to the least disadvantaged, *Listeria* was more common, but *Campylobacter* was less common. In addition to the papers identified by Newman et al. [10], inconsistent results have also been observed among studies that have used syndromic definitions of GI infections, with some reporting higher rates of GI infections among those in lower socioeconomic groups [4, 11, 12] and others observing the opposite [13, 14]. These results clearly demonstrate the disagreements within this area of research.

A number of factors could explain these inconsistent results. The studies identified thus far cover a broad range of pathogens, and it may be that the relationship differs depending on whether the data are analysed at an all-GI-infection, pathogen-specific or species-specific level. This might suggest that the mode of transmission of an organism plays a role in the relationship and that this could be related to potentially socially patterned risk such as rural versus urban residency or exotic foreign travel. Furthermore, these studies have used different study designs, measured SES and GI infection in different ways and controlled for various confounders (such as age, labour market attachment, country of birth and agricultural occupation). Appropriate adjustment of confounding variables requires an understanding of the underlying mechanisms linking SES to GI infection risk, but there is little empirical evidence in this area.

A systematic review is warranted to summarise, organise and make sense of the contradictory findings observed in the literature. Our review aims to build on previous work by exploring the relationship between SES and a full range of GI infections. As it is possible that various socioeconomic or healthcare-seeking behavioural factors could influence whether an individual is diagnosed with a GI infection, we have also included syndromic definitions of GI infections. We aim to explore the current knowledge of the relationship in developed countries; assess the magnitude, statistical significance and direction of the association; and shed light into possible explanations for any observed differences in the risk, incidence or prevalence of GI infections across socioeconomic groups. The results of this review will help to inform the development of empirical research projects by identifying gaps in the literature and areas where further research is required. It will provide evidence of the methods employed previously to investigate the relationship between SES and GI infections, including information on the relevant confounding variables used.

## Methods/design

To improve the transparency and completeness of the protocol, a completed copy of the Preferred Reporting Items for Systematic Reviews and Meta-Analyses for Protocols 2015 (PRISMA-P 2015) checklist [15] can be found in Additional file 1.

### Research question

For individuals from developed countries, is lower compared to higher SES associated with the incidence or prevalence of GI infection?

### Population

Any individual, of any age or gender, from a developed country will be included. A developed country is defined as being a member country of the Organisation for Economic Co-operation and Development (OECD). The OECD aims to continually monitor the economic developments of its 34 member countries and provides policy recommendations to help governments tackle poverty through economic growth and stability [16].

### Exposure

The exposure of interest is lower compared to higher SES, measured at the individual or aggregate level by income, education, occupation, employment or deprivation of area of residence.

### Outcome

The primary outcome of interest will be the incidence or prevalence of any symptomatic GI infection measured using population level surveys, routine surveillance systems, laboratory data or hospitalisation data and includes syndromic definitions of GI infections without a laboratory diagnosis.

### Inclusion/exclusion criteria

Observational studies (cross-sectional, ecological, case-control, cohort [prospective and retrospective]) reporting quantitative results and analysis of empirical data on the prevalence or incidence of any symptomatic GI infection by SES, in a representative population sample, will be included. Socioeconomic status can be measured by occupation, income, education, employment or deprivation at the individual or aggregate level. Only studies conducted in developed countries (defined as being a member country of the OECD), written in or translated into English, reporting on human subjects and using data collected after 1980, will be included. For countries that joined the OECD after 1980, data collection must have occurred after the date the country became a member of the OECD. Studies not meeting the above criteria, including case studies, case series or literature reviews, or studies reporting on outbreaks of GI infection, travel-associated illness only or asymptomatic infections only will be excluded. Studies conducted solely in a specific population subgroup without a general population comparator group or studies conducted in institutional settings such as nurseries, hospitals or the military will be excluded.

### Search strategy

Three search strategies will be used to identify as much relevant literature as possible. Firstly, the electronic searching of three databases will be performed: MEDLINE (Ovid), Scopus and Web of Science Core Collection. The choice of database was discussed with a university librarian, and the three databases chosen were considered most relevant to the research question and likely to yield the highest number of relevant papers.

The search terms were piloted prior to selection and are comprised of specific GI infection and symptom-based terms, socioeconomic and inequality terms, and developed countries of interest (Additional file 2). Relevant synonyms for the SES and GI infection terms were identified using Roget’s Thesaurus online [17] and the thesaurus in MEDLINE by mapping and inspecting the tree for each term. Relevant terms mentioned in articles identified in a pilot search of the literature were also added. Ultimately, the GI infection terms were selected because they represent the main GI pathogens known to cause the greatest burden to public health in the developed world. Whilst not exhaustive, the list is intended to provide a broad spectrum of bacterial, viral and protozoal infections.

The search terms for MEDLINE were developed initially. Where possible, terms were exploded to broaden the search. Terms were added as keywords if they could not be exploded or if the exploded terms were not relevant to the research question. Truncation and proximity operators were also applied as necessary to broaden the search. Terms were combined using Boolean operators.

For consistency, the exact same terms were used for Scopus and Web of Science Core Collection; however, as the functionality of each database is different, it was necessary to adapt the terms developed in MEDLINE for correct use in Scopus and Web of Science Core Collection. Specifically, the terms contained within the exploded terms in MEDLINE needed to be added as individual search terms for use in Scopus and Web of Science Core Collection, and it was necessary to indicate phrases with quotation marks. Additionally, the proximity operators differed for each database.

When the searches are run in Scopus and Web of Science Core Collection, each term will be searched for within the title, abstract and keywords of the documents contained in each database. Filters within the three databases will be applied to restrict the results to publications that have used data from 1980 to the present. As social conditions within countries change over time through development, and methods of classifying SES are also modified over time, restricting to publications using data from 1980 onwards will ensure that the results are as relevant as possible to the present day. Results will also be limited to publications available in the English language. Additionally, where available, filters for ‘human subjects’ and ‘document type’ will be applied to the database search results. All of these filters directly relate to the inclusion criteria. The publications remaining after the filters are applied will then be exported into reference managing software. In this software, the publications from the three databases will be combined and duplicates removed. The remaining publications will then be screened for relevance using the inclusion and exclusion criteria.

Titles and abstracts of the publications will be screened independently by two authors (NA and TR) to ensure consistency in the application of the inclusion and exclusion criteria. Any discrepancies will be discussed and re-examined until an agreement is reached between both reviewers. The full text for studies deemed relevant after title and abstract screening will be retrieved and reviewed in the same way. Where full texts are not available, they will be sought via institutional library sharing agreements. All full-text studies will be screened independently by the same two reviewers to ensure that they conform to the inclusion and exclusion criteria.

The second strategy will consist of searching the reference lists of any studies selected for inclusion in the final review to identify potentially relevant articles that may have been missed by the electronic database searches. The abstracts of any references considered potentially relevant will be sought and screened for inclusion using the pre-defined inclusion and exclusion criteria. The full text for studies deemed relevant after title and abstract screening will be retrieved and reviewed in the same way. This reference list search will be conducted independently by two reviewers (NA and TR), and discrepancies will be discussed and eventually agreed upon at each stage.

The third method will be to conduct a search of the grey literature by entering the terms ‘gastrointestinal infection’ , ‘gastroenteritis’ , ‘diarrhoea’ , ‘diarrhea’ , ‘socioeconomic’ , ‘social class’ , ‘income’ and ‘deprivation’ into the Google internet search engine and the Google Scholar search application and assessing the first 100 results. Each result will be inspected for relevance using the inclusion an exclusion criteria. Again, this will be performed independently by the two reviewers (NA and TR), and disagreement will be resolved through discussion.

### Quality assessment

Risk of bias and quality assessment of the identified studies will be conducted by the review team, independently and then reconciled. The Liverpool University Quality Assessment Tool (LQAT) will be used for this review, which will allow the methodological quality of the studies to be assessed using a tool specific to each study design [18]. It incorporates a star rating system to assess and qualify absence of bias, misclassification and confounding. The LQAT has been used in previous systematic reviews [19, 20] and has been independently evaluated against other quality assessment tools [21]. Any discrepancies between reviewers in the quality assessment of the studies will be discussed and re-examined.

### Data analysis and synthesis

To organise these data and to facilitate comparison, tables will be created by extracting data from each study into a standardised Excel spreadsheet. Data to be extracted will include the following: aim/hypothesis, study design, level of analysis, country, sample size, age, age category, type of GI infection, GI infection method of measurement and data source, measure of SES, SES method of measurement and data source, covariates, statistically significant results, non-significant results, conclusions and quality assessment. Extracted data will be checked for accuracy by at least one other reviewer.

Due to the broad scope of this review, it is anticipated that there will be considerable heterogeneity between studies in terms of design, populations studied and the measurement of primary exposures and outcomes. The synthesis strategy will be driven by the data available; however, to explore the relationship between GI infections and SES, it is anticipated that a subgroup analysis will be performed on study design factors and potential moderating factors of the relationship, including but not limited to the following: pathogen type (based on mode of transmission); age; country (based on climate and relative level of development); methods used to measure GI infection; methods used to measure SES; and level of analysis (aggregate or individual). Separate tables will be created to compare and contrast the results of studies within and between the subgroups. If the data allow, further grouping of the studies within the subgroups will be performed to help summarise the study findings and answer the research question. The LQAT results will be used to determine the strength of the evidence from individual studies, and greater weight will be given to conclusions drawn from the most methodologically robust and reliable studies. A narrative synthesis will help to make sense of what is anticipated to be a diverse body of evidence and may lead to potential explanations for the contrasting findings observed in the literature. The methods used will be written up transparently, and the robustness of the synthesis will be assessed [22].

Where homogenous data allow, meta-analyses will be conducted on combined results. The synthesis strategy outlined above will assist in identifying data suitable for meta-analysis. Heterogeneity will be assessed by examining the forest plots to detect overlapping confidence intervals, using the chi^2^ test with a *P* value of 0.10 to indicate statistical significance, and also applying the *I*^2^ statistic with values of 30 to 60 %, 50 to 90 % and 75 to 100 % used to denote moderate, substantial and considerable levels of heterogeneity, respectively [23]. If the data allow, publication bias will be assessed using a funnel plot, and sensitivity analysis on the basis of study quality will be conducted to explore the robustness of the meta-analysis. RevMan software will be used to conduct these analyses [24]. A ‘Summary of findings’ table [25] will be used to present the results, and the Grading of Recommendations, Assessments, Development and Evaluation approach will be used to assess the quality of the body of evidence [26].

### Dissemination

The systematic review will be submitted for publication. The findings of the review and data will be presented at conferences and will contribute to two PhD projects as part of the National Institute for Health Research Health Protection Research Unit (NIHR HPRU) in Gastrointestinal Infections [27].

## Discussion

Our systematic review aims to provide new insight into the understanding of the mixed results on the relationship between SES and GI infections as suggested by Newman et al. [10], by broadening the focus to a wider range of symptomatic GI infections and exploring whether a more conclusive pattern can be identified. This includes syndromic definitions of GI infections in the absence of laboratory confirmation. By including these definitions, we aim to identify literature on the burden of symptoms by SES and attempt to capture population groups who may not seek healthcare for their illness and consequently may not be included in studies which use laboratory data to identify cases only. This is particularly important for this review as the decision to seek healthcare may be related to SES.

In the UK, it is estimated that 17 million cases of infectious intestinal disease occur every year, resulting in approximately one million general practice consultations [7]. This, coupled with an increasingly overburdened National Health Service (NHS), highlights the importance of understanding the role of SES in GI infections in order to devise policies to target the strata of the population most at risk.

The results of this review will provide a more comprehensive evidence base of the relationship between symptomatic GI infections and SES to inform the development of empirical studies, including effective study design and appropriate data analysis methods, which will be used in two PhD projects.

## Additional files

Additional file 1:
**PRISMA-P 2015 checklist. **The Preferred Reporting Items for Systematic Reviews and Meta-Analyses for Protocols 2015 (PRISMA-P 2015) checklist was used to develop this protocol. Items 1b and 4 were not applicable. Additional file 2:
**Search terms for MEDLINE, Scopus and Web of Science Core Collection. **The search terms that will be used to identify relevant literature across three databases.

## Abbreviations

GI: gastrointestinal
IID: infectious intestinal disease
LQAT: Liverpool University Quality Assessment Tools
NHS: National Health Service
NIHR HPRU: National Institute for Health Research Health Protection Research Unit
OECD: Organisation for Economic Co-operation and Development
PRISMA-P 2015: Preferred Reporting Items for Systematic Reviews and Meta-analyses for Protocols 2015
SES: socioeconomic status
