# Supplementary material for: Relationship between socioeconomic status and gastrointestinal infections in developed countries: a systematic review protocol
Source: Syst Rev. 2016 Jan 21;5:13. doi: 10.1186/s13643-016-0187-7 (PMC4721014; doi:10.1186/s13643-016-0187-7)
Supplement: Additional file 2: — Search terms for MEDLINE, Scopus and Web of Science Core Collection. The search terms that will be used to identify relevant literature across three databases. [file 13643_2016_187_MOESM2_ESM.pdf]

# Appendix A

## *Systematic review search terms*

### MEDLINE (Ovid)

#1. Exp Socioeconomic Factors/
#2. Education*.mp.
#3. Exp Employment/
#4. Income*.mp.
#5. Occupation*.mp.
#6. Poverty.mp.
#7. Poorest.mp.
#8. exp Social Class/
#9. Inequalit*.mp.
#10. Socioeconomic*.mp.
#11. Depriv*.mp.
#12. Disadvantag*.mp.
#13. Salary.mp.
#14. Underprivileged.mp.
#15. Social determinant*.mp.
#16. (Social adj1 factor*).mp
#17. Socio*.mp

#18. 1 or 2 or 3 or 4 or 5 or 6 or 7 or 8 or 9 or 10 or 11 or 12 or 13 or 14 or 15 or 16 or 17

#19. exp Norovirus/
#20. Acute gastroenteritis.mp.
#21. infectious intestinal disease*.mp.
#22. gastrointestinal infection*.mp.
#23. exp Diarrhea/
#24. Rotavirus.mp.
#25. gastrointestinal pathogen*.mp.
#26. gastrointestinal bacteria.mp.
#27. enteric infection*.mp.
#28. diarrh*.mp.
#29. stomach flu.mp.
#30. gastric flu.mp.
#31. stomach bug*.mp.
#32. stomach virus*.mp.
#33. Exp Campylobacter/
#34. Exp Escherichia coli/
#35. Enterobacteriaceae Infection*.mp.
#36. Dysentery, Bacillary.mp
#37. Exp Escherichia coli Infections/
#38. Yersinia enterocolitica.mp.
#39. Exp Salmonella Infections/
#40. Exp Cryptosporidiidae/
#41. Exp Salmonella/

#42. Exp Shigella/
#43. Exp Giardia/
#44. Escherichia coli.mp.
#45. Exp Listeria/
#46. Small round structured virus*.mp.
#47. Winter vomiting disease*.mp.
#48. Sapovirus.mp.
#49. Caliciviridae.mp.
#50. VTEC.mp.
#51. STEC.mp.
#52. exp Foodborne Diseases/
#53. Food poisoning*.mp.
#54. Scombro*.mp.
#55. Clostridium perfringens.mp.
#56. Bacillus cereus.mp.
#57. Hepatitis A.mp.
#58. Hepatitis E.mp.

#59. 19 or 20 or 21 or 22 or 23 or 24 or 25 or 26 or 27 or 28 or 29 or 30 or 31 or 32 or 33 or 34 or 35 or 36 or 37 or 38 or 39 or 40 or 41 or 42 or 43 or 44 or 45 or 46 or 47 or 48 or 49 or 50 or 51 or 52 or 53 or 54 or 55 or 56 or 57 or 58

#60. exp Australia/
#61. exp Austria/
#62. exp Belgium/
#63. exp Canada/
#64. exp Chile/
#65. exp Czech Republic/
#66. exp Denmark/
#67. exp Estonia/
#68. exp Finland/
#69. exp France/
#70. exp Germany/
#71. exp Greece/
#72. exp Hungary/
#73. exp Iceland/
#74. exp Ireland/
#75. exp Israel/
#76. exp Italy/
#77. exp Japan/
#78. exp Korea/
#79. exp Luxembourg/
#80. exp Mexico/
#81. exp Netherlands/
#82. exp New Zealand/
#83. exp Norway/
#84. exp Poland/
#85. exp Portugal/
#86. exp Slovak Republic/
#87. exp Slovenia/
#88. exp Spain/

#89. exp Sweden/
#90. exp Switzerland/
#91. exp Turkey/
#92. exp United Kingdom/
#93. exp United States/

#94. 60 or 61 or 62 or 63 or 64 or 65 or 66 or 67 or 68 or 69 or 70 or 71 or 72 or 73 or 74 or 75 or 76 or 77 or 78 or 79 or 80 or 81 or 82 or 83 or 84 or 85 or 86 or 87 or 88 or 89 or 90 or 91 or 92 or 93

#95. 18 and 59 and 94

| Scopus - TITLE-ABS-KEY | | Web of Science Core Collection - Topic |
|---|---|---|
| #1. | "Career mobility" | |
| #2. | Poverty | |
| #3. | "Social class*" | |
| #4. | "Social mobility" | |
| #5. | Education* | |
| #6. | Employment | |
| #7. | Unemployment | |
| #8. | Income* | |
| #9. | Occupation* | |
| #10. | Poor* | |
| #11. | Inequalit* | |
| #12. | Depriv* | |
| #13. | Disadvantag* | |
| #14. | Salary | |
| #15. | Underprivileged | |
| #16. | "Social determinant*" | |
| #17. | Social pre/1 factor* | Social near/1 factor* |
| #18. | Socio* | |
| #19. | 1 or 2 or 3 or 4 or 5 or 6 or 7 or 8 or 9 or 10 or 11 or 12 or 13 or 14 or 15 or 16 or 17 or 18 | |
| #20. | Norovirus | |
| #21. | "Norwalk virus" | |
| #22. | "Acute gastroenteritis" | |
| #23. | "Infectious intestinal disease*" | |
| #24. | "Gastrointestinal infection*" | |
| #25. | Rotavirus | |
| #26. | "Gastrointestinal pathogen*" | |
| #27. | "Gastrointestinal bacteria" | |
| #28. | "Enteric infection*" | |
| #29. | Diarrh* | |

#30. “Stomach flu”
#31. “Gastric flu”
#32. “Stomach bug*”
#33. “Stomach virus*”
#34. “Escherichia coli”
#35. "Enterobacteriaceae Infection*”
#36. Dysentery Bacillary
#37. “Yersinia enterocolitica”
#38. “paratyphoid fever”
#39. “typhoid fever”
#40. “Small round structured virus*”
#41. “Winter vomiting disease*”
#42. Sapovirus
#43. Caliciviridae
#44. Campylobacter*
#45. Cryptospor*
#46. Salmonell*
#47. Shigell*
#48. Giardia*
#49. Listeri*
#50. VTEC
#51. STEC
#52. “Foodborne Disease*”
#53. Botulism
#54. “Staphylococcal Food Poisoning*”
#55. “Food poisoning*”
#56. Scombro*
#57. “Clostridium perfringens”
#58. “Bacillus cereus”
#59. “Hepatitis A”
#60. “Hepatitis E”

#61. 20 or 21 or 22 or 23 or 24 or 25 or 26 or 27 or 28 or 29 or 30 or 31 or 32 or 33 or 34 or 35 or 36 or 37 or 38 or 39 or 40 or 41 or 42 or 43 or 44 or 45 or 46 or 47 or 48 or 49 or 50 or 51 or 52 or 53 or 54 or 55 or 56 or 57 or 58 or 59 or 60

#62. Australia*
#63. “New South Wales”
#64. “Northern Territory”
#65. Queensland
#66. Tasmania
#67. Victoria
#68. Austria
#69. Belgium
#70. Canada
#71. Alberta
#72. “British Columbia”
#73. Manitoba
#74. “New Brunswick”
#75. “Newfoundland and Labrador”
#76. “Northwest Territories”

#77. “Nova Scotia”
#78. Nunavut
#79. Ontario
#80. “Prince Edward Island”
#81. Quebec
#82. Saskatchewan
#83. “Yukon Territory”
#84. Chile
#85. “Czech Republic”
#86. Denmark
#87. Greenland
#88. Estonia
#89. Finland
#90. France
#91. Paris
#92. Germany
#93. Berlin
#94. Greece
#95. Hungary
#96. Iceland
#97. Ireland
#98. Israel
#99. Italy
#100. Rome
#101. Sicily
#102. Japan
#103. Tokyo
#104. Korea
#105. Seoul
#106. Luxembourg
#107. Mexico
#108. Netherlands
#109. “New Zealand”
#110. Norway
#111. Svalbard
#112. Poland
#113. Portugal
#114. “Slovak Republic”
#115. Slovakia
#116. Slovenia
#117. Spain
#118. Sweden
#119. Switzerland
#120. Turkey
#121. “United Kingdom”
#122. “Great Britain”
#123. “Channel Islands”
#124. Guernsey
#125. England
#126. London
#127. Scotland

#128. Hebrides
#129. Wales
#130. "United States"
#131. "Appalachian Region"
#132. Alabama
#133. Georgia
#134. Kentucky
#135. Maryland
#136. "New York"
#137. Carolina
#138. Ohio
#139. Pennsylvania
#140. Tennessee
#141. Virginia
#142. "Great Lakes Region"
#143. Illinois
#144. Chicago
#145. Indiana
#146. Michigan
#147. Minnesota
#148. Wisconsin
#149. "Mid-Atlantic Region"
#150. Delaware
#151. "District of Columbia"
#152. Baltimore
#153. "New Jersey"
#154. Philadelphia
#155. Iowa
#156. Kansas
#157. Missouri
#158. Nebraska
#159. Dakota
#160. Oklahoma
#161. "New England"
#162. Connecticut
#163. Maine
#164. Massachusetts
#165. Boston
#166. "New Hampshire"
#167. "Rhode Island"
#168. Vermont
#169. Idaho
#170. Montana
#171. Oregon
#172. Washington
#173. Wyoming
#174. "Pacific States"
#175. Alaska
#176. California
#177. "Los Angeles"
#178. "San Francisco"

#179. Hawaii
#180. Arkansas
#181. Florida
#182. Louisiana
#183. “New Orleans”
#184. Mississippi
#185. Arizona
#186. Colorado
#187. Nevada
#188. “New Mexico”
#189. Texas
#190. Utah

#191. 62 or 63 or 64 or 65 or 66 or 67 or 68 or 69 or 70 or 71 or 72 or 73 or 74 or 75 or 76 or 77 or 78 or 79 or 80 or 81 or 82 or 83 or 84 or 85 or 86 or 87 or 88 or 89 or 90 or 91 or 92 or 93 or 94 or 95 or 96 or 97 or 98 or 99 or 100 or 101 or 102 or 103 or 104 or 105 or 106 or 107 or 108 or 109 or 110 or 111 or 112 or 113 or 114 or 115 or 116 or 117 or 118 or 119 or 120 or 121 or 122 or 123 or 124 or 125 or 126 or 127 or 128 or 129 or 130 or 131 or 132 or 133 or 134 or 135 or 136 or 137 or 138 or 139 or 140 or 141 or 142 or 143 or 144 or 145 or 146 or 147 or 148 or 149 or 150 or 151 or 152 or 153 or 154 or 155 or 156 or 157 or 158 or 159 or 160 or 161 or 162 or 163 or 164 or 165 or 166 or 167 or 168 or 169 or 170 or 171 or 172 or 173 or 174 or 175 or 176 or 177 or 178 or 179 or 180 or 181 or 182 or 183 or 184 or 185 or 186 or 187 or 188 or 189 or 190

#192. 19 and 61 and 191
